# A Novel DFT-Based DOA Estimation by a Virtual Array Extension Using Simple Multiplications for FMCW Radar

**DOI:** 10.3390/s18051560

**Published:** 2018-05-14

**Authors:** Bongseok Kim, Sangdong Kim, Jonghun Lee

**Affiliations:** Advanced Radar Technology Laboratory (ART Lab.), Daegu Gyeongbuk Institute of Science and Technology (DGIST), Daegu 42988, Korea; remnant@dgist.ac.kr (B.K.); kimsd728@dgist.ac.kr (S.K.)

**Keywords:** FMCW, virtual array, DOA estimation, DFT

## Abstract

We propose a novel discrete Fourier transform (DFT)-based direction of arrival (DOA) estimation by a virtual array extension using simple multiplications for frequency modulated continuous wave (FMCW) radar. DFT-based DOA estimation is usually employed in radar systems because it provides the advantage of low complexity for real-time signal processing. In order to enhance the resolution of DOA estimation or to decrease the missing detection probability, it is essential to have a considerable number of channel signals. However, due to constraints of space and cost, it is not easy to increase the number of channel signals. In order to address this issue, we increase the number of effective channel signals by generating virtual channel signals using simple multiplications of the given channel signals. The increase in channel signals allows the proposed scheme to detect DOA more accurately than the conventional scheme while using the same number of channel signals. Simulation results show that the proposed scheme achieves improved DOA estimation compared to the conventional DFT-based method. Furthermore, the effectiveness of the proposed scheme in a practical environment is verified through the experiment.

## 1. Introduction

Recently, there have been several studies on frequency modulated continuous wave (FMCW) radar systems due to its many advantages, including lower cost and complexity, over equivalent pulse radar systems [1,2,3,4]. In FMCW radar systems, estimating the direction of arrival (DOA) is a major research issue. In DOA estimations, discrete Fourier transform (DFT)-based DOA estimation, or so-called “DBF (digital beam forming)”, is usually employed [1]; the approach has the advantage of low complexity for real-time signal processing compared to the super resolution algorithms, such as multiple signal classifier (MUSIC) and the estimation of signal parameters via rotational invariance technique (ESPRIT), which has very high complexity [5,6,7]. One of the main challenges in DOA estimations based on DFT is to enhance the resolution of the DOA estimation while reducing the missing detection probability [1,8]. In order to achieve these goals, it is essential to have a considerable number of channel signals. In practice, however, it is very difficult to increase the number of channel signals because of the constraints of space and cost.

As a solution, virtual array (VA) methods have been proposed [9,10,11,12]. By using mathematical manipulations or multiple frequency bands, these methods try to increase the resolution of DOA estimation using the existing channel signals. In [9,10], by employing conjugate counterparts of the channel signals, a VA was formed to extend the equivalent array aperture, so that it could handle more sources than the given channel signals. In order to maintain the phase information even after the conjugate operation, non-circular signals were assumed, such as binary phase shift keying (BPSK) modulated signals. However, as noted in [11], the assuming of a non-circular signal is not practical. This is because, even if the source is non-circular, the received signal, due to phase shifts, is not real-valued but complex-valued, which can be different for each signal. Meanwhile, in [12], the authors tried to increase the resolution by using multiple frequency bands, that is, they exploited differences in phase shift according to frequency band. In practice, however, it is not easy to use multiple frequency bands, due to the high cost of multiple radio frequency (RF) devices. Meanwhile, DOA estimation algorithms using multiple input multiple output (MIMO) antenna systems have been proposed in order to improve resolution [13,14]. Without large increasing the physical size of system, these algorithms can improve the DOA resolution by virtually increasing channel signals. However, these algorithms also require not only the additional transmit antennas, but also additional resources such as time and frequency for orthogonality between transmit signals.

In this paper, we propose a novel DFT-based DOA estimation method by exploiting the point that extrapolation is possible by using the given signal in the assumption of sinusoid signal as in [15,16]. In order to increase the number of channel signals, virtual channel signals are generated by using multiplications of the given channel signals, without the assumption of a non-circular signal and using of additional transmit antennas. In this way, the proposed scheme significantly reduces the probability of missing detection and root mean square error (RMSE), compared with the conventional DFT-based DOA estimation scheme, while using the same number of channel signals. Despite the improved performance, the computation complexity of the proposed scheme is almost identical to that of the conventional DFT-based DOA estimation scheme. When there are multiple targets, interference terms inevitably occur due to the cross term of the multiplications during the generation of virtual channel signals. We investigate the effect of these interference terms on the overall performance. Simulation results show that the proposed scheme achieves a lower RMSE and a lower probability of missing detection than those of the conventional scheme. Furthermore, the effectiveness of the proposed scheme is verified by experiments in a practical environment.

## 2. Signal Model and Notation

**Figure 1:**
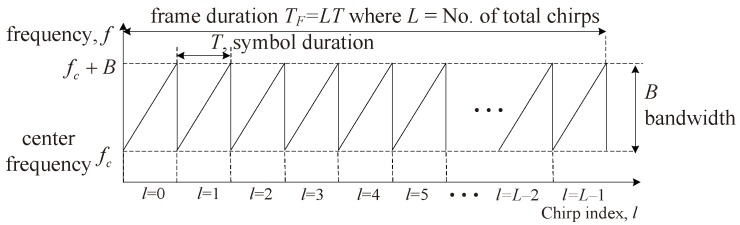
Structure of the transmit (TX) signal of FMCW radar where *f* is frequency, *B* is bandwidth, *L* is the number of chirps per one frame, *T* is symbol duration, $f_{c}$ is center frequency, and $T_{F}$ is frame duration.

As shown in Figure 1, the transmitted (TX) FMCW signal frame, which is composed of a total of *L* chirps, is denoted by x(t) and expressed as

(1) $$\begin{array}{c} x\left(t\right)=\sum_{l=0}^{L-1}x_{0}\left(t\right)\prod \left(t-lT\right), \end{array}$$

where ∏(t) is the normalized rectangular signal and *T* is the duration of an FMCW chirp signal $x_{0}\left(t\right)$. An FMCW chirp signal $x_{0}\left(t\right)$ is expressed as follows:

(2) $$\begin{array}{c} x_{0}\left(t\right)=\exp\left(j2\pi \left(f_{c}t+\frac{\mu }{2}t^{2}\right)\right), \end{array}$$

where $f_{c}$ is the carrier frequency and μ is the rate of change of the instantaneous frequency of a chirp signal.

We consider *M* far-field, non-coherent, narrow-band targets impinging on a uniform linear array (ULA) with *K* elements. The receive (RX) signal of the *k*th array element for the *l*th chirp is denoted by $r_{l,k}\left(t\right)$ and is expressed as [2]:

(3) $$\begin{array}{c} r_{l,k}\left(t\right)=\sum_{m=1}^{M}{\dot{a}}_{m}x_{0}\left(t-\tau_{m}\right)\exp\left(j2\pi f_{D,m}Tl\right)\exp\left(j\frac{2\pi }{\lambda }d_{s}k\sin\theta_{m}\right)+{\dot{w}}_{l,k}\left(t\right)\;\;\mathrm{for}\;k=1,\ldots ,K, \end{array}$$

where ${\dot{a}}_{m}$ is the complex amplitude of the *m*th target, $d_{s}$ is the spacing between the adjacent elements, λ is the wavelength of the carrier frequency, and $\tau_{m}$, $f_{D,m}$, and $\theta_{m}$ are the round trip time delay, Doppler frequency due to velocity of moving target, and DOA elements of the *m*th target, respectively; ${\dot{w}}_{l,k}\left(t\right)$ is the additive white Gaussian noise (AWGN) signal at the *k*th array and the *l*th chirp. By multiplying the conjugated FMCW TX signal $x_{0}{\left(t\right)}^{*}$ by $r_{l,k}\left(t\right)$ and assuming $d_{s}=\lambda /2$, the beat signal for the *l*th chirp and the *k*th array $y_{l,k}\left(t\right)$ is obtained and expressed as the product of the time-of-arrival (TOA), Doppler and DOA terms as follows:

(4) $$\begin{array}{ccc} y_{l,k}\left(t\right) & = & r_{l,k}\left(t\right)\times x_{0}{\left(t\right)}^{*}\\ & = & \sum_{m=1}^{M}\underset{≜{\tilde{a}}_{m}}{\underset{︸}{{\dot{a}}_{m}\exp\left(-j\left(2\pi f_{c}\tau_{m}-\mu \tau_{m}^{2}/2\right)\right)}}\underset{\;\mathrm{TOA}\;\mathrm{term},≜\eta_{m}\left(t\right)}{\underset{︸}{\exp\left(-j2\pi \mu \tau_{m}t\right)}}\underset{\mathrm{Doppler}\;\mathrm{term},\;≜v_{m}^{l}}{\underset{︸}{\exp\left(j2\pi f_{D,m}Tl\right)}}\underset{\mathrm{DOA}\;\mathrm{term},\;≜z_{m}^{k}}{\underset{︸}{\exp\left(j\pi k\sin\theta_{m}\right)}}\\ & + & \underset{\mathrm{noise}\;\mathrm{term},\;≜{\tilde{w}}_{l,k}}{\underset{︸}{{\dot{w}}_{l,k}\left(t\right)x_{0}{\left(t\right)}^{*}}}\\ & = & \sum_{m=1}^{M}{\tilde{a}}_{m}\eta_{m}\left(t\right)v_{m}^{l}z_{m}^{k}+{\tilde{w}}_{l,k}\left(t\right). \end{array}$$

After the analogue to digital conversion (ADC) of $y_{l,k}\left(t\right)$, the discrete time model of Equation (4) with the sampling frequency $f_{s}$ is denoted by $y_{l,k}\left[n\right]$ i.e., $y_{l,k}\left[n\right]=y_{l,k}\left(nT_{s}\right)$ for $n=0,1,\ldots ,N_{s}-1$, where $T_{s}$ is the sampling interval, i.e., $T_{s}=1/f_{s}$, $N_{s}$ is the number of samples, i.e., $N_{s}=T/T_{s}$, and thus Equation (4) is rewritten as:

(5) $$\begin{array}{c} y_{l,k}\left[n\right]=\sum_{m=1}^{M}{\tilde{a}}_{m}\eta_{m}\left[n\right]v_{m}^{l}z_{m}^{k}+{\tilde{w}}_{l,k}\left[n\right]. \end{array}$$

## 3. DFT-Based DOA Estimation in FMCW Radar Systems

**Figure 2:**
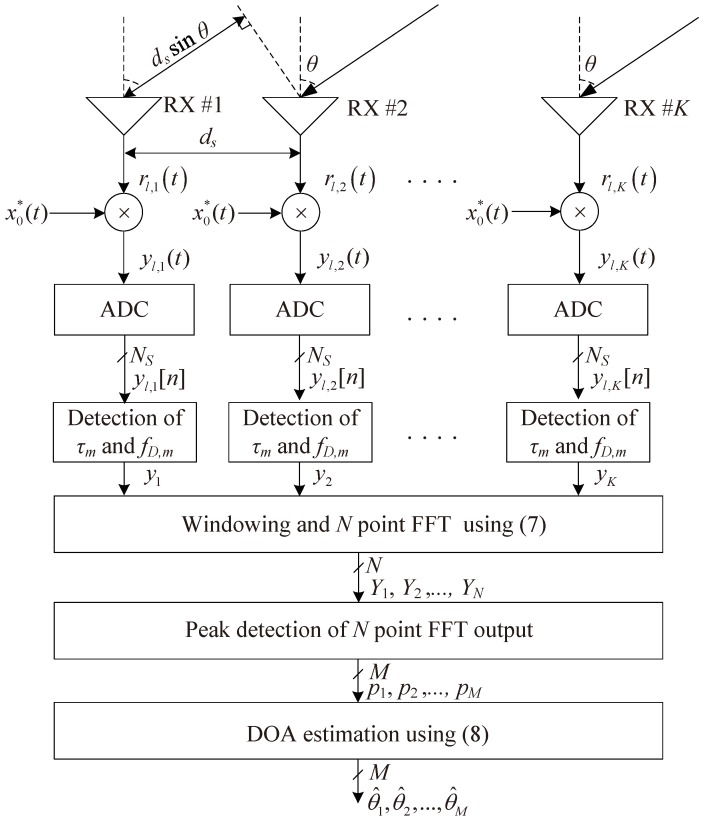
Structure of direction of arrival (DOA) estimation in FMCW radar.

This section addresses DFT-based DOA estimation in FMCW radar systems. Figure 2 shows the structure of DOA estimation of FMCW radar systems. As shown in Figure 2, first, the TOA term to estimate the range of the target and the Doppler term to estimate the speed of the target are estimated using 2D DFT [4]. However, estimation of the TOA and the Doppler terms is not a major issue in this paper and thus we omit a detailed description of them. In order to focus on DOA estimation, the product of ${\tilde{a}}_{m}\left[n\right]$ and TOA and Doppler terms is expressed as a new variable, $a_{m}\left[n\right]$, i.e., $a_{m}\left[n\right]={\tilde{a}}_{m}\eta_{m}\left[n\right]v_{m}$. In addition, by omitting sample index *n* and chirp index *l*, Equation (4) is simply expressed as follows:

(6) $$\begin{array}{c} y_{k}=\sum_{m=1}^{M}a_{m}z_{m}^{k}+{\tilde{w}}_{k}. \end{array}$$

In order to estimate DOA information from Equation (6), DFT for DBF is performed, that is, the DFT operation is performed on $y_{k}$ for 1≤k≤K [8]. The *q*th DFT output of $y_{k}$ is denoted by $Y_{q}$ and obtained as follows:

(7) $$\begin{array}{c} Y_{q}=\sum_{k=1}^{K}y_{k}W_{N}^{q(k-1)}\;\;\mathrm{for}\;\;1\leq q\leq N, \end{array}$$

where $W_{N}$ is the *N* point DFT operator, i.e., $W_{N}=\exp\left(-j2\pi /N\right)$. Then, peak detection processing is performed, that is, the *m*th peak index $p_{m}$ corresponding to the *M* peaks in $|Y_{q}|$ is obtained for *m* = 1, …, *M*. From the obtained $p_{m}$, finally, the *m*th estimated DOA term ${\hat{\theta }}_{m}$ is estimated as follows:

(8) $$\begin{array}{c} {\hat{\theta }}_{m}=\sin^{-1}\left(\frac{2}{N}\left(p_{m}-\left(\frac{N}{2}+1\right)\right)\right). \end{array}$$

## 4. Proposed Algorithm

This section illustrates the proposed DFT-based DOA estimation. The key idea of the proposed scheme is to extend the effective number of channel signals by using multiplication of the given real channel signals. First, we address the structure of the proposed algorithm. Then, we investigate the effect caused by the interference terms that occur due to multiplication when there are multiple targets; we show that the proposed scheme improves the DOA resolution.

### 4.1. Structure of the Proposed Algorithm

In this section, we illustrate the structure of the proposed algorithm. The proposed algorithm newly generates the *k*th channel signal $u_{k}$ by multiplication among the given real channel (beat) signals in Equation (6) for two intervals as follows:

(9) $$\begin{array}{c} u_{k}=\left\{\begin{array}{c} y_{1}y_{k}\;\;\mathrm{for}\;1\leq k\leq K,\\ y_{K}y_{k-K+1}\;\mathrm{for}\;K+1\leq k\leq 2K-1. \end{array}\right. \end{array}$$

**Figure 3:**
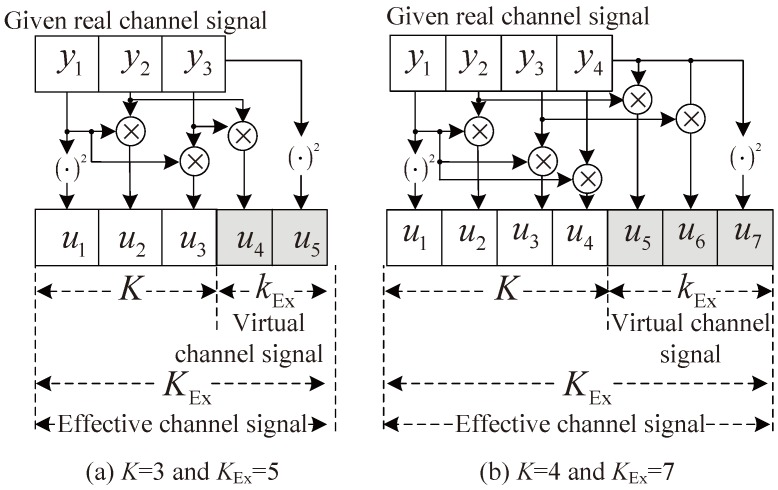
Structure of the proposed algorithm with *K* = 3 and 4.

Figure 3 shows the structures of the proposed scheme with K=3 and K=4, respectively. In Figure 3, $k_{\mathrm{Ex}}$ denotes the number of virtual channel signals and $K_{\mathrm{Ex}}$ is the number of effective channel signals, i.e., $K_{\mathrm{Ex}}=K+k_{\mathrm{Ex}}$. That is, $k_{\mathrm{Ex}}$ can be maximally set to K−1 and the maximum $K_{\mathrm{Ex}}$ can be set to 2K−1. In Figure 3a,b, it can be seen that two and three virtual channel signals (the shadowed part) are additionally generated, respectively.

In order to investigate the features of the proposed algorithm, let us observe a new virtual signal $u_{k}$ obtained by multiplication of $y_{p}$ and $y_{k},$ where $y_{p}$ is equal to $y_{1}$ for 1≤k≤K and $y_{p}$ is equal to $y_{K}$ for K+1≤k≤2K−1. Therefore, the newly generated channel signal $u_{k}$ is expressed as:

(10) $$\begin{array}{ccc} u_{k} & = & y_{p}y_{k}\\ & = & \underset{\mathrm{desired}\;\mathrm{term}≜d_{k}}{\underset{︸}{\sum_{m=1}^{M}a_{m}^{2}z_{m}^{k+1}}}+\underset{\mathrm{interference}\;\mathrm{term}≜i_{k}}{\underset{︸}{\sum_{m\neq p}^{M}a_{m}z_{m}^{p}\sum_{p\neq m}^{M}a_{p}z_{p}^{k}}}+\underset{\mathrm{noise}\;\mathrm{term}≜w_{k}}{\underset{︸}{{\tilde{w}}_{p}\sum_{m=1}^{M}a_{m}z_{m}^{k}+{\tilde{w}}_{k}\sum_{m=1}^{M}a_{m}z_{m}^{p}+{\tilde{w}}_{p}{\tilde{w}}_{k}}}\\ & = & d_{k}+i_{k}+w_{k}, \end{array}$$

where $d_{k}$ is the desired term, which includes the DOA information of each target, $i_{k}$ is the interference term due to multiplication among multiple signals, and $w_{k}$ is the noise term. Meanwhile, $w_{k}$ has statistics identical to the case before multiplication. This is because the two noise terms ${\tilde{w}}_{p}\sum_{m=1}^{M}a_{m}z_{m}^{k}$ and ${\tilde{w}}_{k}\sum_{m=1}^{M}a_{m}z_{m}^{p}$ are linear combinations of each noise component, and thus the two noise terms still hold on to a Gaussian distribution. In addition, since the distribution of the multiplication of complex Gaussian random variables is Gaussian, ${\tilde{w}}_{p}{\tilde{w}}_{k}$ also follows a complex Gaussian distribution [17]. Hence, the noise terms can be denoted as one variable, i.e., $w_{k}$, as shown in Equation (10). For easy understanding, we show an example in the case of K=2 and M=1. By using Equation (9), $u_{1}$, $u_{2}$, and $u_{3}$ are obtained as follows:

(11) $$\begin{array}{c} u_{1}=y_{1}\times y_{1}=a^{2}\exp\left(j\pi \sin\theta \right)+w_{1}, \end{array}$$

(12) $$\begin{array}{c} u_{2}=y_{1}\times y_{2}=a^{2}\exp\left(j2\pi \sin\theta \right)+w_{2}, \end{array}$$

(13) $$\begin{array}{c} u_{3}=y_{2}\times y_{2}=a^{2}\exp\left(j3\pi \sin\theta \right)+w_{3}. \end{array}$$

From Equations (11)–(13), not only amplitude term is maintained as $a^{2}$ for *k* = 1, 2 and 3, but also the order of theta increases according to the array index *k*. It implies that the additional channel signal with DOA information is virtually generated by the proposed algorithm.

In order to estimate DOA information $\theta_{m}$ in Equation (10), the DFT operation for DBF is performed on $u_{k}$ for $1\leq k\leq K_{\mathrm{Ex}}$, as shown in the previous section. Therefore, the *q*th DFT output is denoted by $U_{q}$ and obtained as follows:

(14) $$\begin{array}{c} U_{q}=\sum_{k=1}^{K_{\mathrm{Ex}}}u_{k}W_{N}^{q(k-1),} \end{array}$$

where $W_{N}$ is the *N* point DFT operator, i.e., $W_{N}=\exp\left(-j2\pi /N\right)$. Then, as shown in Figure 2, peak detection processing is performed in the proposed scheme. Thus, the *M* indices corresponding to the *M* peaks in $|U_{q}|$ are obtained. Finally, the DOA terms are estimated from the obtained *M* indices.

**Figure 4:**
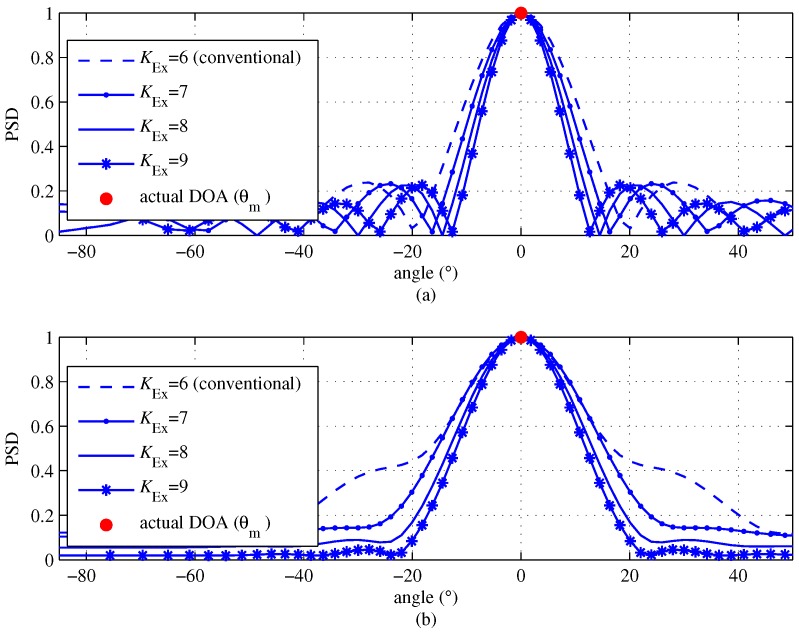
Discrete Fourier transform results of the proposed scheme according to the number of effective channel signals $K_{\mathrm{Ex}}$ for a single target with K=6, signal to noise ratio (SNR) = 10 dB and N=64 (**a**) without Hanning windowing; (**b**) with Hanning windowing.

In order to effectively show the feasibility of the proposed scheme, Figure 4 shows that $|U_{q}|$ is obtained by the proposed scheme for several $K_{\mathrm{Ex}}$s with signal to noise ratio (SNR) = 10 dB, N=64 and *K* = 6 for single target condition. For the single target condition, there is no interference term, i.e., $i_{k}=0$ in Equation (10), and thus there is only the desired term and the noise term. In Figure 4, it can be seen that as $K_{\mathrm{Ex}}$ increases, the magnitude of the DFT output for estimating the DOA terms gets sharper. This is because the number of channel signals virtually increases from *K* to $K_{\mathrm{Ex}}=2K-1$, as shown in Equations (9) and (14). Meanwhile, as shown in Equation (10), the interference term $i_{k}$ coexists in multiple targets environment, but we will illustrate in the next section that the effect by interference is insignificant. From the results shown in Figure 4, therefore, we can conjecture that the proposed scheme will achieve a better DOA estimation than the conventional DOA-DFT based estimation scheme. Figure 4b shows the DFT result of the proposed algorithm with Hanning windowing in order to mitigate the effect of sidelobe. Compared with Figure 4a, the width of the DFT result becomes broadened, but the effect of sidelobe is significantly reduced due to Hanning windowing.

### 4.2. Analysis of Interference Signal Due to Cross Terms

This section provides an analysis of the interference signal $i_{k}$, which is inevitably generated by the multiplication in Equation (10). The DFT is a linear operation, as is well known. Hence, we can focus on just the interference term $i_{k}$. According to Equation (9), $i_{k}$ for the first interval, 1≤k≤K is denoted by $i_{k}^{\left(1\right)}$ and is expressed as follows:

(15) $$\begin{array}{ccc} i_{k}^{\left(1\right)} & = & a_{1}z_{1}\left(a_{2}z_{2}^{k}+a_{3}z_{3}^{k}+\ldots +a_{M}z_{M}^{k}\right)+a_{2}z_{2}\left(a_{1}z_{1}^{k}+a_{3}z_{3}^{k}+\ldots +a_{M}z_{M}^{k}\right)+\ldots \\ & = & a_{1}z_{1}^{k}\left(a_{2}z_{2}+a_{3}z_{3}+\ldots \right)+a_{2}z_{2}^{k}\left(a_{1}z_{1}+a_{3}z_{3}+\ldots \right)+a_{3}z_{3}^{k}\left(a_{1}z_{1}+a_{2}z_{2}+\ldots \right)+\ldots \\ & = & \sum_{m=1}^{M}\alpha_{m}z_{m}^{k}, \end{array}$$

where $\alpha_{m}=a_{m}\sum_{m^{\prime }\neq m}^{M}a_{m^{\prime }}z_{m^{\prime }}$. Using an expression similar to Equation (15), $i_{k}$ for the second interval K+1≤k≤2K−1 is denoted by $i_{k}^{\left(2\right)}$ and expressed as follows:

(16) $$\begin{array}{c} \begin{array}{ccc} i_{k}^{\left(2\right)} & = & a_{1}z_{1}^{k-K+1}\left(a_{2}z_{2}^{K}+a_{3}z_{3}^{K}\right)+a_{2}z_{2}^{k-K+1}\left(a_{1}z_{1}^{K}+a_{3}z_{3}^{K}\right)+a_{3}z_{3}^{k-K+1}\left(a_{1}z_{1}^{K}+a_{2}z_{2}^{K}\right)+\ldots \\ & = & \sum_{m=1}^{M}\beta_{m}z_{m}^{k-K+1}, \end{array} \end{array}$$

where $\beta_{m}=a_{m}\sum_{m^{\prime }\neq m}^{M}a_{m^{\prime }}z_{m^{\prime }}^{K}$. Consequently, $i_{k}^{\left(1\right)}$ and $i_{k}^{\left(2\right)}$ are generally expressed as:

(17) $$\begin{array}{c} i_{k}^{\left(1\right)}=\left\{\begin{array}{c} \sum_{m=1}^{M}\alpha_{m}z_{m}^{k},\;\;\mathrm{for}\;1\leq k\leq K,\\ \;\;\;0,\;\;\;\;\;\;\;\;\;\;\;\mathrm{for}\;K+1\leq k\leq 2K-1, \end{array}\right. \end{array}$$

(18) $$\begin{array}{c} i_{k}^{\left(2\right)}=\left\{\begin{array}{c} \;\;\;0,\;\;\;\;\;\;\;\;\;\;\;\;\;\;\;\mathrm{for}\;1\leq k\leq K,\\ \sum_{m=1}^{M}\beta_{m}z_{m}^{k-K+1},\;\mathrm{for}\;K+1\leq k\leq 2K-1. \end{array}\right. \end{array}$$

For a more intuitive understanding, $i_{k}^{\left(2\right)}$ is rewritten in a form similar to $i_{k}^{\left(1\right)}$, in the following manner:

(19) $$\begin{array}{c} i_{k}^{\left(2\right)}=\sum_{m=1}^{M}\beta_{m}z_{m}^{k+1},\;\;\mathrm{for}\;1\leq k\leq K-1. \end{array}$$

**Figure 5:**
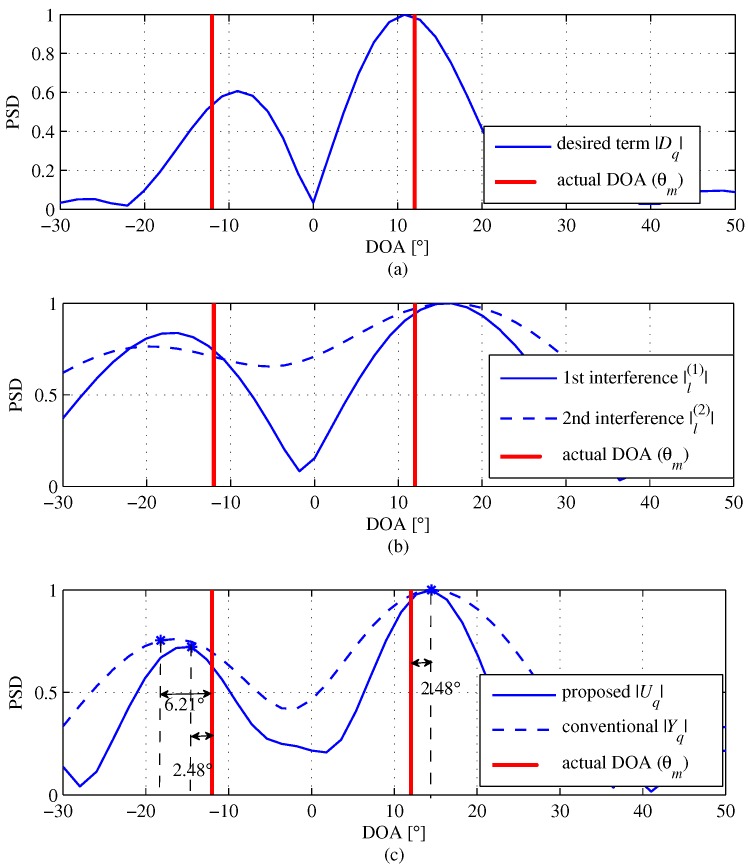
Snapshots of DFT outputs of the proposed algorithm with $K=5\;(K_{\mathrm{Ex}}=9)$, SNR = 10 dB and M=2 to observe the effect due to interference terms on the overall system performance (**a**) comparison between the desired term and actual DOA; (**b**) comparison between interference terms and actual DOA; (**c**) comparison between the proposed and the conventional algorithms.

From Equation (17) and Equation (19), it is shown that both of $i_{k}^{\left(1\right)}$ and $i_{k}^{\left(2\right)}$ contain DOA terms $z_{m}^{k}$ and $z_{m}^{k+1}$, respectively. Comparing $d_{k}$ in Equation (10) and $i_{k}^{\left(1\right)}$ in Equation (17) and $i_{k}^{\left(2\right)}$ in Equation (19), $d_{k}$ and $i_{k}^{\left(1\right)}$ and $i_{k}^{\left(2\right)}$ have the same DOA information $z_{m}$ with different amplitude. As is well known, DFT is a linear operator and thus it is implied that $i_{k}^{\left(1\right)}$ and $i_{k}^{\left(2\right)}$ are insignificant to the overall performance. In order to confirm our assumption that interference components will not have a significant impact on overall performance, we analyze the DFT output of $u_{k}$. By using the linearity of DFT, $U_{q}$ in Equation (14) can be expressed as the sum of the desired and interference terms denoted by $D_{q}$, $I_{q}^{\left(1\right)}$ and $I_{q}^{\left(2\right)}$, as follows:

(20) $$\begin{array}{ccc} U_{q} & = & \underset{≜D_{q}}{\underset{︸}{\sum_{k=1}^{2K-1}d_{k}W_{N}^{q(k-1)}}}+\underset{≜I_{q}^{\left(1\right)}}{\underset{︸}{\sum_{k=1}^{2K-1}i_{k}^{\left(1\right)}W_{N}^{q(k-1)}}}+\underset{≜I_{q}^{\left(2\right)}}{\underset{︸}{\sum_{k=1}^{2K-1}i_{k}^{\left(2\right)}W_{N}^{q(k-1)}}}, \end{array}$$

where the noise term is omitted for simplicity. Figure 5 provides snap shots of the normalized magnitude of the DFT outputs with K=5, SNR = 10 dB, $K_{\mathrm{Ex}}=9$ and M=2. In Figure 5a, the result of the extended desired signal $D_{q}$ is shown. The extended desired signal $D_{q}$ include not only noise terms and but also interference terms. The peaks of $|D_{q}|$ almost precisely follow the actual DOA terms. Figure 5b shows the results of $I_{q}^{\left(1\right)}$ and $I_{q}^{\left(2\right)}$. From Figure 5b, it is not as much as $|D_{q}|$, but the peak of two interference terms follows the actual DOA terms well as expected. Figure 5c shows the result of $U_{q}$; the peaks of $U_{q}$ also follow the actual DOA terms even though $U_{q}$ includes interference terms as well as noise terms compared to the conventional algorithm. That is, the estimation error by the proposed algorithm is about 2.48°, which is smaller than the estimation error of 6.21° by the conventional algorithm, as shown in Figure 5c. This implies that the interference signal is insignificant to the overall performance.

**Figure 6:**
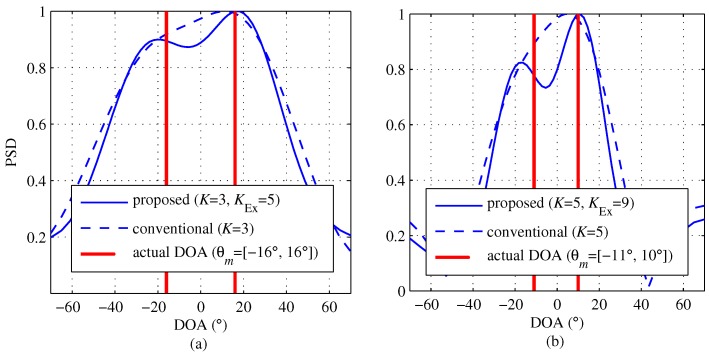
Comparison of the resolution of proposed ($U_{q}$) and conventional ($Y_{q}$) algorithms with M=2, (**a**) K=3 and [$\theta_{1}$, $\theta_{2}$] = [$-16^{{}^{\circ}}$, $16^{{}^{\circ}}$]; (**b**) K=5 and [$\theta_{1}$, $\theta_{2}$] = [$-11^{{}^{\circ}}$, $10^{{}^{\circ}}$].

In Figure 6, the results of DFT output of the proposed and conventional algorithms are shown in order to compare the resolution performance between two algorithms in the case of M=2. Figure 6 shows that the proposed algorithm distinguishes between the two different DOA terms. On the other hand, in the conventional algorithm, two targets are merged to be unity and thus as if it is shown that there is a single target.

## 5. Simulation Results

In this section, we provide simulation results to evaluate the performance of the proposed scheme. Results of the proposed scheme were compared with results from a conventional scheme based on Equation (6). Commonly, we set $d_{s}=\lambda /2$ and $f_{c}=$ 24 GHz and the complex amplitude $a_{m}$ in Equation (4) was randomly and independently generated with uniform distribution, that is, the magnitude and angle of $a_{m}$ are $0\leq |a_{m}|\leq 1$ and $0\leq ∡a_{m}\leq 2\pi$, respectively. The size of DFT *N* was set to 64. We present the Monte Carlo simulation results averaged over $10^{5}$ estimates. In order to effectively compare the performance of the proposed and the conventional schemes, we employ two indicators, i.e., the root mean square error (RMSE) and the probability of missing detection, denoted by $P_{\mathrm{miss}}$. RMSE is defined as $\mathrm{RMSE}=\sqrt{\frac{1}{M\times 10^{5}}\sum_{i=1}^{10^{5}}\sum_{m=1}^{M}{\left(\theta_{m}-{\hat{\theta }}_{m}\right)}^{2}}$ and $P_{\mathrm{miss}}$ means the probability that the number of peaks obtained by peak detection of DFT output will be less than the number of targets. If the number of peaks found by peak detection is lower than the number of targets *M*, we do not include this case in the RMSE evaluation. Meanwhile, the angular difference between the targets is set by considering the angular resolution, Δθ in given condition. The angular resolution Δθ is approximately calculated as follows [18,19]:

(21) $$\begin{array}{c} \Delta \theta \approx \frac{0.886\lambda }{Kd_{s}\cos\theta }. \end{array}$$

**Figure 7:**
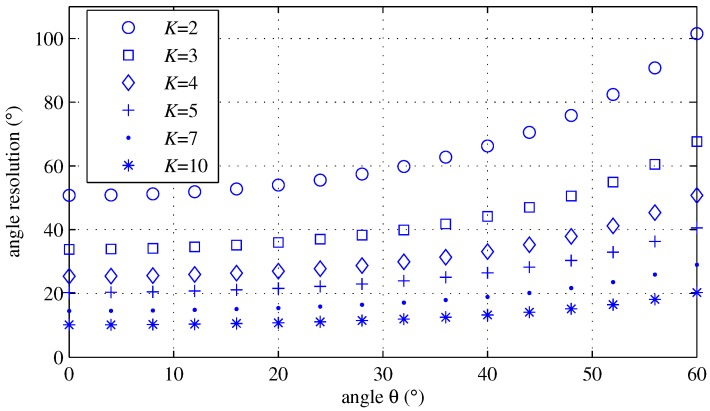
Angle resolution Δθ according to angle for several *K* values.

Figure 7 shows Δθ according to *K* and angles using Equation (21). The angle resolution Δθ becomes smaller as *K* increases and θ approaches zero. In performing the simulation, information of the angle resolution will be used.

**Figure 8:**
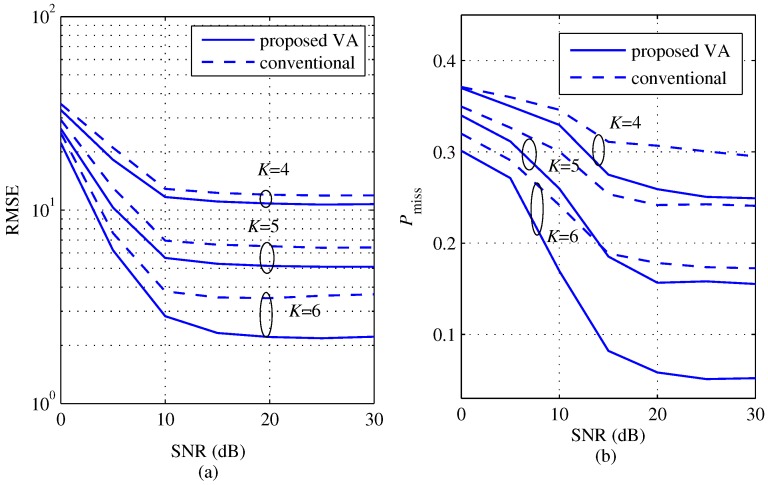
Root mean square error (RMSE) and probability of missing detection comparisons between the proposed and the conventional algorithms according to SNR for several *K*s with M=2 ($\theta_{1}=-10^{{}^{\circ}}$, $\theta_{2}=11^{{}^{\circ}}$) and $K_{\mathrm{Ex}}$ = 2K−1, (**a**) RMSE; (**b**) probability of missing detection.

Figure 8 shows a comparison between the proposed and the conventional algorithms of the RMSE and $P_{\mathrm{miss}}$ according to SNR for several *K*s with M=2 ($\theta_{1}=-10^{{}^{\circ}}$, $\theta_{2}=11^{{}^{\circ}}$). In the proposed algorithm, $K_{\mathrm{Ex}}$ is set to 2K−1. Two angles $\theta_{1}$ and $\theta_{2}$ are set such that $|\theta_{1}-\theta_{2}|=21^{{}^{\circ}}$ by considering angle resolution Δθ at *K* = 5. From Figure 8a,b, the RMSE and $P_{\mathrm{miss}}$ of both the proposed and the conventional schemes commonly decrease as *K* and SNR increase. However, observing more closely, the proposed scheme achieves lower RMSE and lower $P_{\mathrm{miss}}$ than the conventional scheme for all *K* values and all SNR regions. Moreover, the difference of the performance between the two schemes as *K* increases. Consequently, these results imply that the proposed scheme overcomes the limitation of the conventional scheme by virtually increasing the number of effective channel signals.

**Figure 9:**
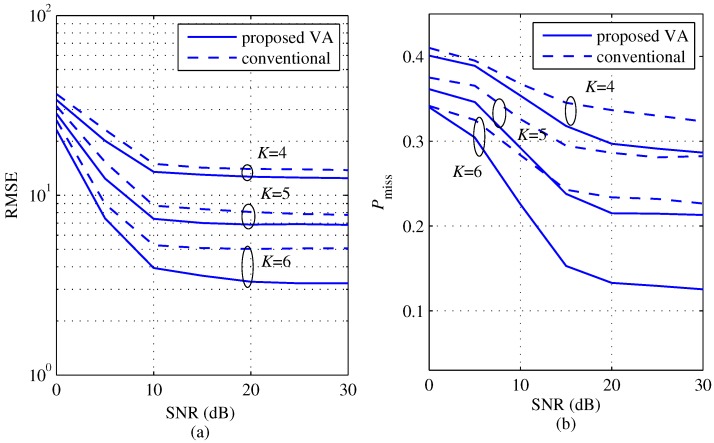
RMSE and probability of missing detection comparisons between the proposed and the conventional algorithms according to SNR for several *K*s with M=2 ($\theta_{1}=-10^{{}^{\circ}}$, $\theta_{2}=8^{{}^{\circ}}$) and $K_{\mathrm{Ex}}$ = 2K−1; (**a**) RMSE; (**b**) probability of missing detection.

**Figure 10:**
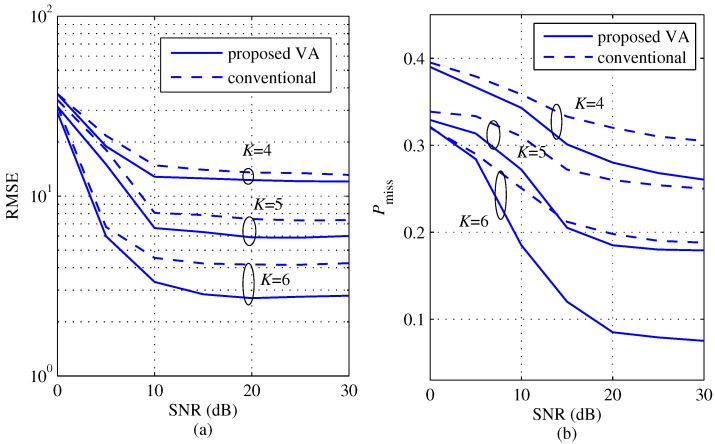
RMSE and probability of missing detection comparisons between the proposed and the conventional algorithms according to SNR for several *K*s with M=2 ($|\theta_{1}-\theta_{2}|=21^{{}^{\circ}}$), $K_{\mathrm{Ex}}$ = 2K−1 and the center of angles $\in [-30^{{}^{\circ}},30^{{}^{\circ}}]$, (**a**) RMSE; (**b**) probability of missing detection.

Figure 9 shows a comparison of the RMSE and $P_{\mathrm{miss}}$ between the proposed and the conventional algorithms according to SNR for several *K*s with M=2 $(\theta_{1}=-10^{{}^{\circ}}$, $\theta_{2}=8^{{}^{\circ}})$ in order to evaluate the improvement by the proposed algorithm in the case when the angle difference is smaller than the angle resolution in Equation (21) , i.e., $|\theta_{1}-\theta_{2}|<\Delta \theta$. Meanwhile, Figure 10 shows a comparison of the RMSE and $P_{\mathrm{miss}}$ between the proposed and the conventional algorithms according to SNR for several *K*s with M=2 ($|\theta_{1}-\theta_{2}|=21^{{}^{\circ}}$) and center of two targets $\in [-30^{{}^{\circ}},30^{{}^{\circ}}]$) in order to observe effect due to the center of two targets. The center is set randomly and independently generated with uniform distribution within $\left[-30^{{}^{\circ}},30^{{}^{\circ}}\right]$. From the results of Figure 9 and Figure 10, both of the RMSE and $P_{\mathrm{miss}}$ of the two schemes are larger than the results of Figure 8. These results mean that the performances of two schemes degrade due to the angle resolution and the center of targets compared to the results of Figure 8. However, the proposed scheme achieves lower RMSE and lower $P_{\mathrm{miss}}$ than the conventional scheme for all *K* values even in these cases.

**Figure 11:**
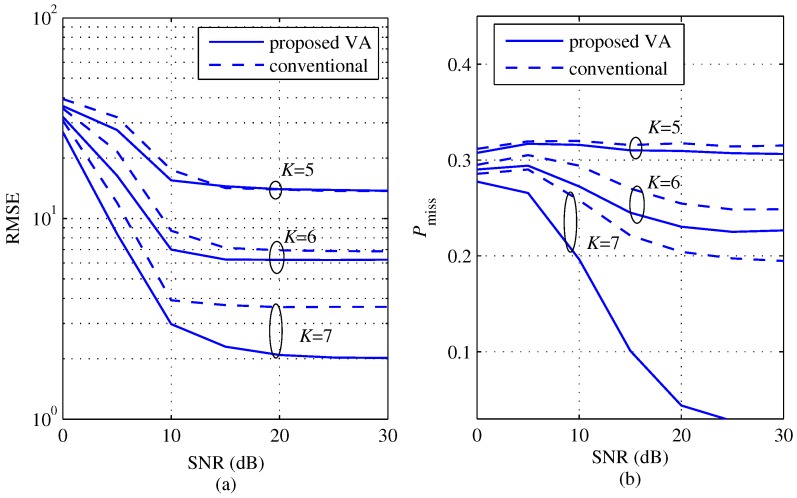
RMSE and probability of missing detection comparisons between the proposed and the conventional algorithms according to SNR for several *K*s with M=3 ($\theta_{1}=-21^{{}^{\circ}}$, $\theta_{2}=-2^{{}^{\circ}}$, $\theta_{3}=17^{{}^{\circ}}$), (**a**) RMSE; (**b**) probability of missing detection.

Figure 11 shows a comparison of RMSE and $P_{\mathrm{miss}}$ according to SNR for several *K*s with M=3 ($\theta_{1}=-21^{{}^{\circ}}$, $\theta_{2}=-2^{{}^{\circ}}$, $\theta_{3}=17^{{}^{\circ}}$). Similar to the results in Figure 8, the values of RMSE and $P_{\mathrm{miss}}$ of the two schemes commonly decrease as *K* and SNR increase. Even in the case of M=3, we can still observe that the proposed scheme improved the performance compared to the conventional scheme.

## 6. Experiments

In order to confirm the effectiveness of the proposed VA algorithm in a practical environment, we perform the experiments inside an anechoic chamber, located at the Daegu Gyeongbuk Institute of Science and Technology (DGIST) in Korea. This section consists of two subsections. First, equipment for experiments is addressed; and we then deal with the experimental results.

### 6.1. Experimental Setup

**Figure 12:**
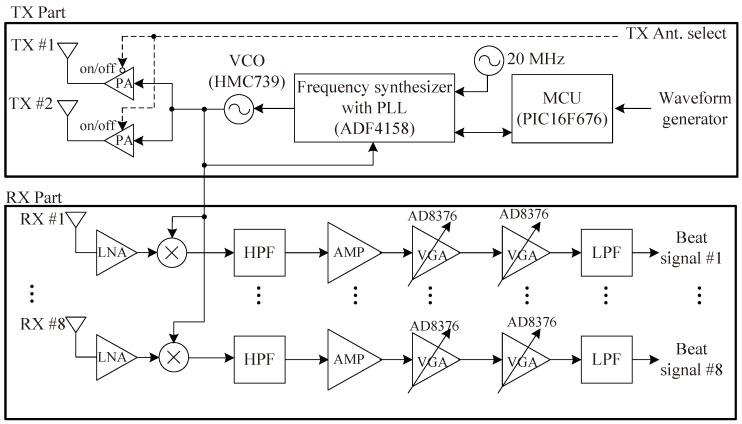
Block diagram of the 24 GHz radar radio frequency (RF) module.

**Figure 13:**
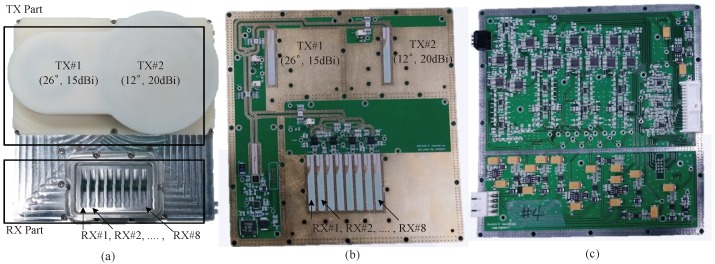
Images of 24 GHz 2 × 8 RF module (front-end module) (**a**) outside; (**b**) top-view; (**c**) bottom-view.

**Figure 14:**
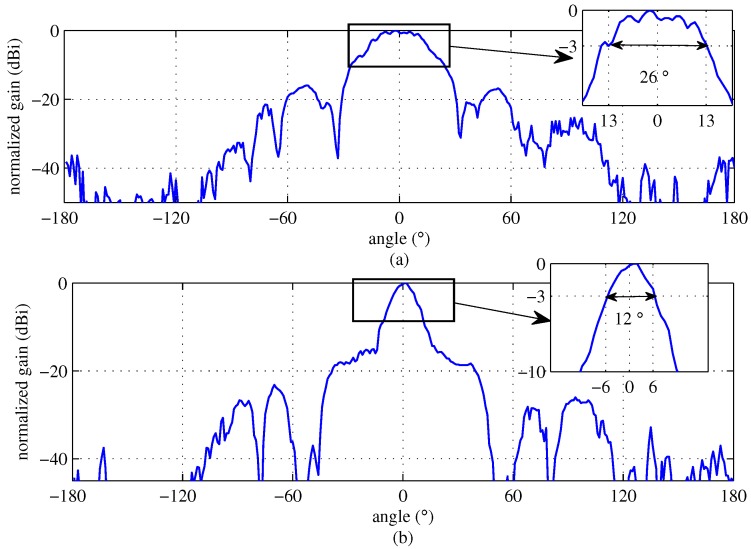
Radiation pattern of TX antennas; (**a**) TX antenna 1 that can cover 26°; (**b**) TX antenna 2 that can cover 12°.

We employed the 24 GHz FMCW radar system, which has two TX antennas and eight RX antennas, as designed in [20]. Figure 12 shows a block diagram of the RF module, i.e., the front end module (FEM); Figure 13 shows outside, top-view and bottom-view images of the RF module. As shown in Figure 12 and Figure 13, the RF module is composed of TX and RX parts. The TX part includes the micro controller unit (MCU), frequency synthesizer with PLL (phase-locked loop), and voltage-controlled oscillator (VCO). The MCU controls the frequency synthesizer with PLL (ADF4158). The VCO output is finally connected to the two TX antennas through the power amplifier (PA). In this system, two TX antennas can not be used simultaneously and thus, one TX antenna in two TX antennas should be selected. As shown in Figure 12, one TX antenna is selected by the TX antenna selection signal (dashed line). The radiation pattern of TX antennas is shown in Figure 14. The azimuth angles of the TX antennas are 26° and 12° according to the beam-width corresponding to the 3 dB gain, as shown in Figure 14 [20]. These results mean TX antenna 1 can cover an azimuth angle of 26° and TX antenna 2 can cover an azimuth angle of 12° . Therefore, we choose TX antenna 1 because the azimuth angle to be measured in the next section is more than 25°. In the RX part, there are the eight RX antennas, the low noise amplifiers (LNAs), high pass filters (HPFs), variable gain amplifier (VGA), and low pass filters (LPFs). The outputs of the LNAs are multiplied to TX signals and they then pass the HPFs with 150 KHz of bandpass frequency. HPF is employed to remove the DC-offset component due to the direct conversion receiver of FMCW radar system [21,22]. The outputs of HPFs are amplified by amplifiers with 6 dB gain and VGAs with −2.5 dB to 42.5 dB gain and then the eight channels beat signals are obtained after amplified signals pass the LPFs with 1.7 MHz of bandpass frequency. The noise figure of RX is 8.01 dB and the RX antenna gain is 10 dB. The RX antenna azimuth beamwidth is 99.6° and elevation beamwidth is 9.9° [20].

**Figure 15:**
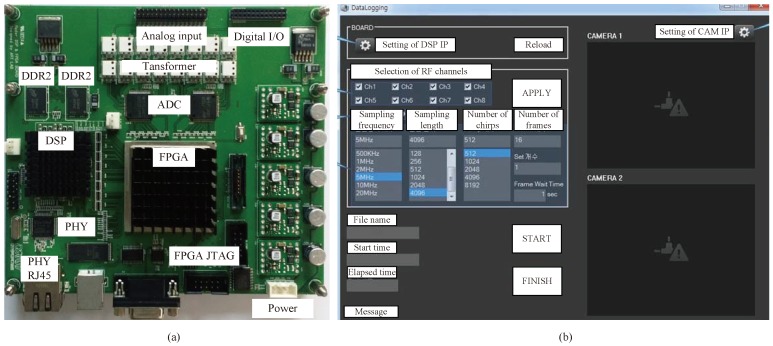
Back-end module (BEM) system for experiment; (**a**) data logging board; (**b**) graphic user interface (GUI).

Figure 15 shows the back end module (BEM) system for the experiment. As shown in Figure 15, BEM is composed of a data logging board and graphic user interface (GUI) software. In Figure 15a, the data logging board includes digital signal processing (DSP) and a field programmable gate array (FPGA) operating at up to 1 GHz. The analog signal is converted to digital data at up to eight channels, with 20 MHz sampling rate through the ADC. The two 2 GB DDR2 SDRAMs are external memories of the DSP, providing a total of 512 Mbytes of data storage space. When the external memory is filled, the data is transferred to the computer through the Ethernet. In Figure 15b, GUI software provides the convenience of logging board settings. By using the GUI, we can set the desired RF channel, sampling frequency, sampling length, the number of chirps, the number of frames, and so on. In addition, BEM can be easily started and terminated, and IP (internet protocol) settings for communication with a PC are also possible through GUI software.

**Figure 16:**
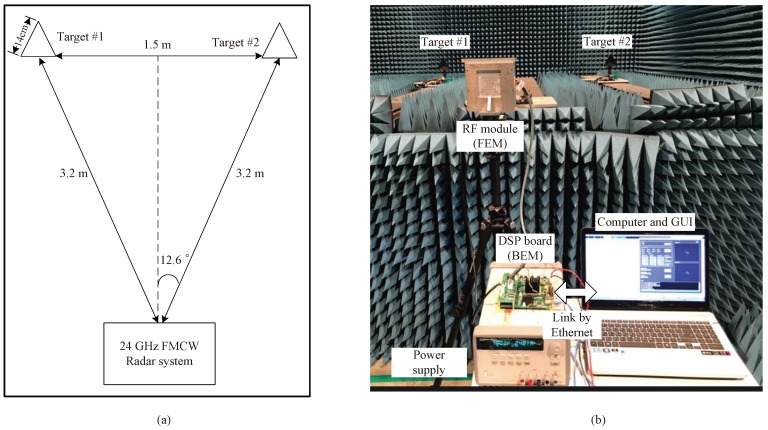
Experiment scenario and real image; (**a**) experiment scenario; (**b**) real image of experiment in chamber.

Figure 16 shows a scenario and real image of an experiment in the chamber. As shown in Figure 16a, two targets are set at the same distance from the radar in order to focus on measuring of DOA. As can be seen in Figure 16b, the experiment was performed inside an anechoic chamber, located at DGIST in Korea, in order to avoid the negative effects due to the undesired echoes. This chamber is designed for 8 GHz to 110 GHz and its size is 5 m (W) × 10 m (L) × 4 m (H). The duration of the chirp (ramp) *T* is set to 400 μs, bandwidth is set to 1 GHz and the sampling frequency is set to 5 MHz. The corner-reflectors with a side length of 14 cm is employed as targets in order to avoid the decrease of radar cross section (RCS). The number of chirps per one frame is set to 256 and the number of frames is set to 64. In the previous step, 2048 point FFT was performed for the range estimation and 256 point FFT was performed in the DOA estimation step.

### 6.2. Experiment Results

**Figure 17:**
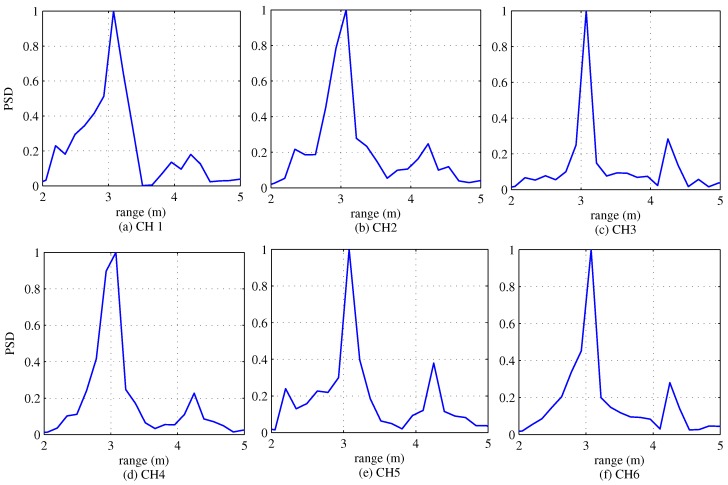
Experiment results of range detection for each channel.

This section addresses experiment results in order to verify the improvement induced by the proposed algorithm. Figure 17 shows the range estimation results from channel 1 to channel 6. In this experiment, speed estimation is not included because the two targets are stopped and thus the speeds of the two targets are zero. According to these experiment results, a range of about 3.07 m is estimated by performing DFT in the sample index domain. This implies that the experiment results for the range estimation are the almost same as the actual range of 3.2 m. In order to detect DOA terms, we use only the range bins corresponding to 3.07 m.

**Figure 18:**
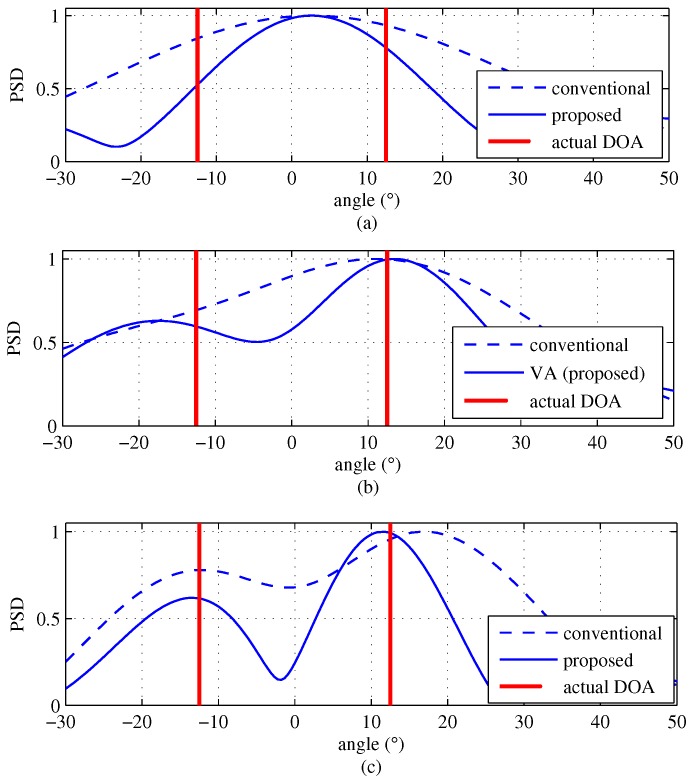
Experiment results with *M* = 2 ($\theta_{1}=-12.6{}^{\circ}$, $\theta_{2}=12.6{}^{\circ}$), (**a**) number of arrays K=3; (**b**) number of arrays K=4; (**c**) number of arrays K=5.

Figure 18 shows the experiment results with K=3, K=4 and K=5 for the scenario shown in Figure 16a. In Figure 18a, in the case of K=3, it can be seen that neither the proposed scheme nor the conventional scheme can distinguish between the two targets, even though there are two targets due to the lack of channels. Meanwhile, as shown in Figure 18b, in the case of K=4, while the conventional scheme can not distinguish between the two targets, the proposed scheme distinguishes the two targets. This result shows that the proposed scheme achieves an improvement in resolution due to the virtually increased number of channels. In Figure 18c, in the case of K=5, both the proposed and the conventional schemes can distinguish between the two targets because the two schemes no longer lack a sufficient number of channels. According to these results, it is concluded that the proposed scheme can effectively improve the angle resolution by increasing the number of channels.

## 7. Conclusions

We have proposed an improved DFT-based DOA estimation scheme by virtually increasing the number of channel signals. We showed how the number of channels can be increased by using simple multiplication. By showing that DFT output was sharpened due to the virtually increased number of channels, we provided a reason for the improved resolution. In addition, in order to determine whether the interference terms were insignificant in the multiple targets condition, we mathematically investigated that the interference terms were insignificant because the interference terms had the same DOA information as the desired signals. Simulation results showed that the proposed scheme achieved a lower RMSE and lower missing detection probability than those of the conventional DOA estimation algorithm using DFT. Furthermore, the effectiveness of the proposed scheme was verified by experiment in a practical environment.
